# A prolonged innate systemic immune response in COVID-19

**DOI:** 10.1038/s41598-022-13986-5

**Published:** 2022-06-15

**Authors:** Sandra Ekstedt, Krzysztof Piersiala, Marianne Petro, Agneta Karlsson, Åsa Kågedal, Susanna Kumlien Georén, Lars Olaf Cardell

**Affiliations:** 1 Division of ENT Diseases, Department of Clinical Science, Intervention and Technology, Karolinska Institutet, Stockholm, Sweden; 2 Department of ENT Diseases, Karolinska University Hospital, Stockholm, Sweden

**Keywords:** Innate immune cells, Innate immunity, Immunology, Diseases

## Abstract

Despite the introduction of vaccines, COVID-19 still affects millions of people worldwide. A better understanding of pathophysiology and the discovery of novel therapies are needed. One of the cells of interest in COVID-19 is the neutrophil. This cell type is being recruited to a site of inflammation as one of the first immune cells. In this project, we investigated a variety of neutrophils phenotypes during COVID-19 by measuring the expression of markers for migration, maturity, activation, gelatinase granules and secondary granules using flow cytometry. We show that neutrophils during COVID-19 exhibit altered phenotypes compared to healthy individuals. The activation level including NETs production and maturity of neutrophils seem to last longer during COVID-19 than expected for innate immunity. Neutrophils as one of the drivers of severe cases of COVID-19 are considered as potential treatment targets. However, for a successful implementation of treatment, there is a need for a better understanding of neutrophil functions and phenotypes in COVID-19. Our study answers some of those questions.

## Introduction

Coronavirus disease 2019 (COVID-2019) caused by severe acute respiratory syndrome coronavirus 2 (SARS-CoV-2) has, since it was first reported in December 2019, affected millions of people worldwide and has had a huge impact on public health, economies, and population overall well-being. Despite vaccines being available in some countries, there are still thousands of people worldwide choosing not to get vaccinated or not having access to vaccines. These people have an increased risk of getting infected as well as dying from COVID-19. The introduced mRNA and vector vaccines prevent severe disease and hospitalization, however, there is still a lack of a definite antiviral or immune therapy that would help to manage the disease in those who develop the severe acute respiratory syndrome.

Neutrophils, which are the most abundant type of innate immune cells in the human body, have recently emerged as one of the most important drivers of the hyperinflammation leading to acute respiratory distress in COVID-19^1^. One of the hallmarks of innate immune reactions is that it is believed to last no longer than 96 h and be the first line of defense against non-specific agents. Neutrophils’ main function is an immediate immunological defense against bacterial and fungal pathogens. The role of neutrophils in viral diseases is not fully understood. The evidence suggests that neutrophils during viral infection act indirectly as immunomodulating cells and directly by cytokine secretion, virus internalization and production of neutrophil extracellular traps (NETs)^2,3^. Furthermore, RSV and human cytomegalovirus (HCMV) have been shown to prolong the survival of neutrophils by delaying apoptosis^4,5^ and to increase the production of mucus, leading to obstruction of the lungs^6^. Neutrophils may even act as antigen‐presenting cells during the antiviral immune response and thus, act as immune response regulators^7^.

Numerous studies have shown an association between neutrophil numbers and a worse prognosis for patients infected with SARS-CoV-2^8,9^. The neutrophil-to-lymphocyte ratio (NLR), a simple ratio between the neutrophil and lymphocyte counts measured in peripheral blood, has been shown to be increased in patients who develop acute respiratory distress syndrome (ARDS) and have a high risk for death in course of COVID-19. Neutrophils are increased in numbers not only in peripheral circulation but have also been found abundantly in lung tissue during autopsies of patients who died of COVID-19^10^. Furthermore, several studies reported increased levels of NETs markers and neutrophil associated cytokines that correlated with poorer prognosis in COVID-19 patients^11^.

Undoubtedly, recent research demonstrates that neutrophils are important players in the pathophysiology of COVID-19, predominantly associated with progression to severe illness and poor prognosis. For this reason, neutrophils gained interest as a potential target to treat severe cases of COVID-19^8^. Nevertheless, to succeed with the design of novel therapeutics, there is a need to address some of the still unanswered questions regarding the role of neutrophils during SARS-CoV-2 infection. To answer some of them, we designed this project where we investigated the phenotypes, activation markers, NETs and secretion of cytokines of neutrophils in patients diagnosed with COVID-19.

## Methods

### Patient characteristics

Patients admitted to our COVID-19 subunit at the ENT department of Karolinska University Hospital, Huddinge, with a positive PCR test for COVID-19 were eligible for inclusion. Blood was collected at one-time point. A healthy control group was also recruited, and blood was collected. The study was approved by the Swedish Ethical Review Authority in Gothenburg (Number 2020-02579). All procedures performed in studies involving human participants were in accordance with the ethical standards of the institutional and national research committee and with the 1964 Helsinki Declaration and its later amendments or comparable ethical standards. All participants provided written informed consent before any procedures.

### Antibodies

Antibodies from BD Biosciences; CD15-BV650 (#563142, clone W6D3), CD45-PE-CF594 (#562279 clone HI30), CD16-V450 (#560474 clone 3G8), CD62L-BV510 (#563203 clone DREG-56), CD47-PE (#556046 clone B6H12), CD36-APC (#550956 clone CB38), CD11b-APC-Cy7 (#557754 clone ICRF44), CD66b-PerCP-Cy5.5 (#562254 clone G10F5), CD49d-BV711(#563177 clone 9F10), CD43-BB515 (#564542 clone 1G10) and CD24-PE-Cy7 (#561646 clone ML5).

### Sample preparation and Flow cytometry

Whole blood was collected in heparin tubes. The red blood cells were removed with ammonium chloride erythrocyte lysis solution (0.8 mM NH4Cl, 10 mM KHCO3 0.1 mM EDTA). The remaining cells were then washed once with PBS and incubated with antibodies (CD15, CD45, CD16, CD62L, CD47, CD36, CD11b, CD66b, CD49d, CD43 and CD24) at room temperature in the dark for 20 min and washed with PBS. Cells were resuspended in PBS with 1% paraformaldehyde (HistoLab #02178) and analyzed on LSR FORTESSA × 20 (BD Biosciences). Analysis of the flow cytometry data was performed with FlowJo version 10.7.1 (LLC, USA). The gating strategy for identification of neutrophils is summarized in Supplementary Fig. 1. Plasma was saved at − 80 °C until analyzed.

### Data processing

FACS3.0 files were imported into FlowJo software version 10.6.2 (LLC, USA). FlowAI were run on all events before gating. Cells were first gated based on side scatter (SSC-A) and forward scatter (FSC-A) to exclude debris. Doublets were excluded and neutrophils were defined as CD45 + and CD15 + cells. Traditional gating was then applied to evaluate different markers on the neutrophils and study migration, maturity, activation, gelatinase granules and secondary granules.

The downsample plugin (v.3.3) and concentration tool was used to visualize multiparametric data from a comparable number of CD15 + cells from each patient group. Dimensionality reduction was performed using the t-SNE tool in FlowJo (v.10.6.2). The following parameters were used to create t-SNE plots: iterations = 1000, perplexity = 30, learning rate (eat) = 5040, gradient algorithm—Barnes-Hut. Clusters of phenotypically related cells were then detected by Phenograph (v.3.0). Phenograph was run with K = 100. The following markers were used to delineate the t-SNE and perform Phenograph clustering: CD16, CD62L, CD47, CD36, CD11b, CD66b, CD49d, CD43 and CD24.

### Measurements of cfDNA, DNA-histone complex, TNFα, IL-6 and IL-8

The presence of NETs was assessed by measuring both the concentration of cell-free DNA (cfDNA) using an InvitrogenTM QubitTM 3 Fluorometer (Thermo Fisher Scientific), and the DNA-histone complexes levels (cell-free nucleosomes) using the Human Cell Death Detection ELISA PLUS (Roche, Switzerland). TNFα, IL-6 and IL-8 was measured in plasma samples with ELISA (human TNF-a Quantikine HS ELISA Kit, R&D systems, sensitivity: 4.32 pg/ml, Human IL-6 Quantikine ELISA Kit R&D systems, sensitivity: 0.7 pg/mL, Human IL-8/CXCL8 Quantikine ELISA Kit, R&D systems, sensitivity: 7.5 pg/mL).

### Statistical analysis

Statistical analyses were performed with GraphPad Prism version 9.2 (GraphPad Software, La Jolla, CA, USA). A D'Agostino & Pearson omnibus normality test was used to determine if the data were normally distributed. For more than two sets of data, a two-way ANOVA with Bonferroni post-hoc test was performed. For two sets of data, the Mann–Whitney test was performed when non-normally distributed data and the unpaired T-test when normally distributed data. The outcome was presented as the mean ± SEM or as median (IQR).

## Results

### Patient characteristics

Twenty-seven hospitalized patients with PCR verified SARS-CoV-2 infection were enrolled in the study. The median age of the covid patients was 66 years (range of 48–90 years). There were 17 male (63%) and 10 female (37%) participants. The control group consisted of 6 male (50%) and 6 female (50%) healthy participants verified with PCR negative test for SARS-CoV-2. The median age of the covid patients was 33 years (range of 25–58 years). Demographic and clinical characteristics of enrolled patients and healthy controls are summarized in Table 1.

**Table 1:** Summary of the clinical and demographic characteristics of enrolled patients and healthy controls.

	COVID-19 (n = 27)	Healthy controls (n = 12)
Age (median; range)	67 years (48–90)	33 years (25–58)
**Sex (n, %)**		
Male	17 (63.0)	6 (50.0)
Female	10 (37.0)	6 (50.0)
Days of hospitalization (median; range)	8 (2–66)	n/a^d^
**Comorbidities (n, %)**		n/a^d^
Diabetes	4 (14.8)	
Hypertension	11 (40.7)	
COPD^a^	2 (7.4)	
Asthma	1 (3.7)	
**Smoking (n, %)**		n/a
Yes	3 (11.1)	
No	24 (88.9)	
**Clinical laboratory parameters (mean, ± SD)**		n/a
CRP^b^	62.06 ± 57.75	
Procalcitonin	0.205 ± 0.29	
WBCs^c^	7.88 ± 2.502	

^a^Chronic obstructive pulmonary disease.

^b^C-reactive protein.

^c^White blood cell count.

^d^Not applicable.

### COVID-19 is characterized by neutrophil hyperreactivity and high production of pro-inflammatory cytokines

**Figure 1:**
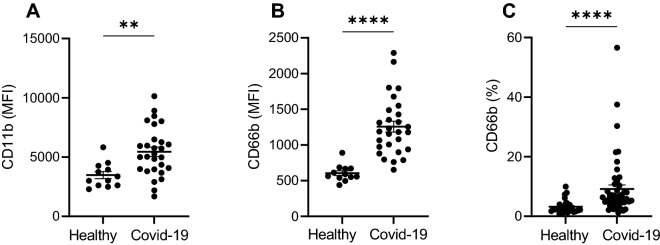
Expression of neutrophil activation markers seen during COVID-19. MFI is significantly increased in COVID-19 patients for CD11b (**A**) and CD66b (**B**). A significantly higher percentage of neutrophils in COVID-19 express on its surface CD66b (**C**).

**Figure 2:**
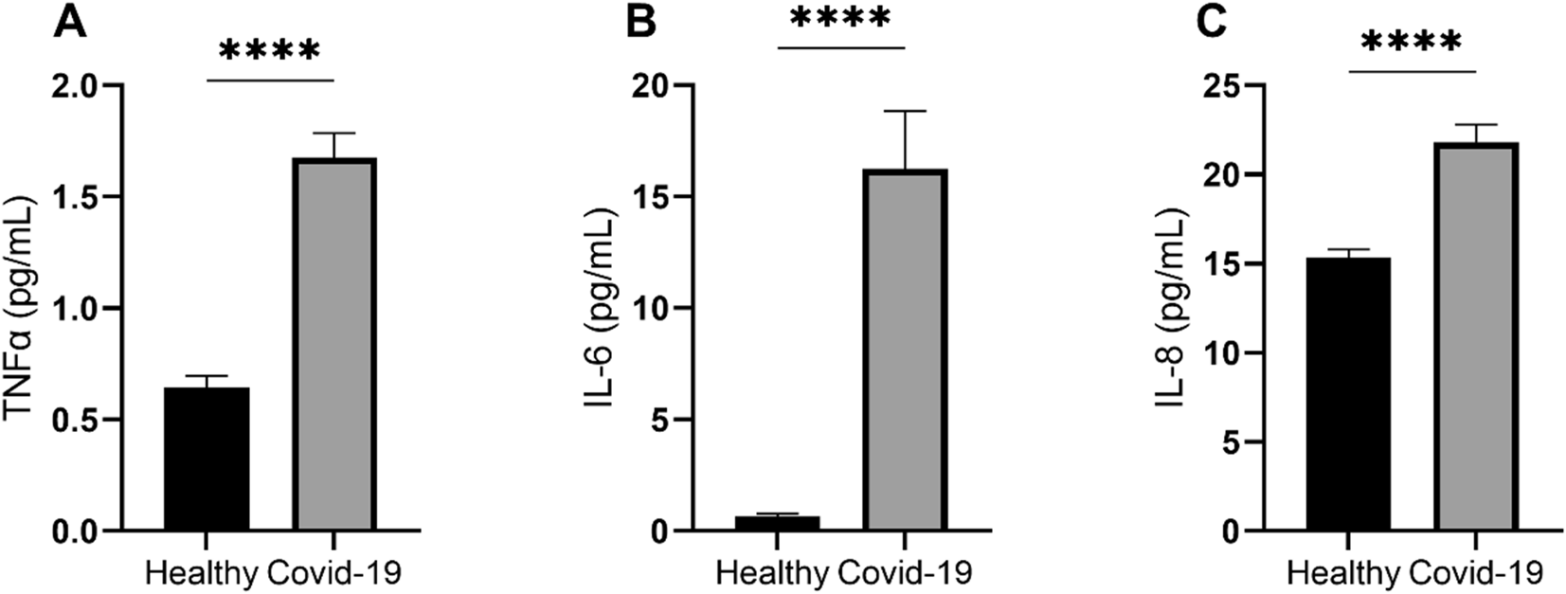
The level of TNFα (**A**), IL-6 (**B**) and IL-8 (**C**) in plasma is increased during COVID-19 infection compared with healthy individuals.

Neutrophils from patients with COVID-19, in comparison to healthy individuals, were characterized by significantly higher expression intensity of neutrophil activation marker CD11b (mean fluorescence intensity (MFI) ± SD of 5448 ± 2071 and 3475 ± 1035, respectively; *p* < 0.01; Fig. 1A). The intensity of fluorescence and percentage of neutrophils positive for granulocyte activation marker CD66b was also significantly increased in COVID-19 patients compared with controls (1257 ± 408 and 605 ± 115.7, respectively; *p* < 0.0001; Fig. 1B and median and interquartile range of 6.220 (4.6–10.3) compared with 2.380 (1.83–3.98), respectively; *p* < 0.0001; Fig. 1C). Furthermore, we found that the plasma level of the pro-inflammatory cytokines, TNF-α, IL-6 and IL-8,—central cytokines in antiviral responses and cytokines driving a phenomenon of cytokine storm—was significantly higher in patients with COVID-19 compared to the healthy donors (TNF, median and interquartile range of 1.545 (1.165–2.076) compared with 0.6383 (0.513–0.730); *p* < 0.0001; Fig. 2A; IL-6, median and interquartile range of 8.749 (3.79–21.79) compared with 0.4955 (0.01–0.819); *p* < 0.0001); Fig. 2B; IL-8, median and interquartile range of 20.19 (17.47–24.07) compared with 15.07 (13.62–16.30); *p* < 0.0001); Fig. 2C).

### Neutrophils in COVID-19 patients show increased survival compared to neutrophils from healthy donors

**Figure 3:**
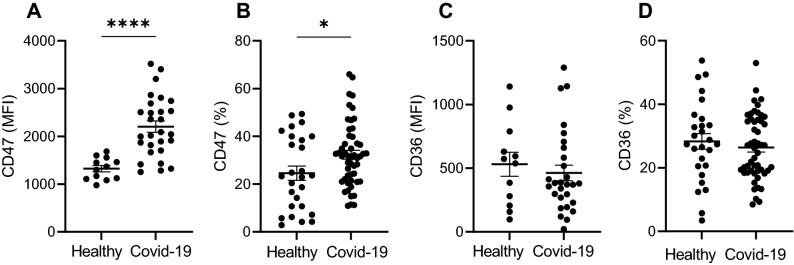
Expression of ”eat me” and ”don’t eat me” markers on neutrophils. Neutrophils from patients with COVID-19 infection show increased expression of ”don’t eat me” marker CD47 (**A**,**B**). Whereas, ”eat me” marker CD36 is expressed on comparable levels in both COVID-19 patients and healthy controls (**C**,**D**).

Neutrophil populations detected in blood of COVID-19 patients showed, in comparison to healthy donors, significantly higher expression of CD47, which is a molecular “don’t eat me” signal that inhibits phagocytosis of the expressing cell and prolongs its survival (MFI ± SD of 2204 ± 631 and 1323 ± 231, respectively; *p* < 0.0001; Fig. 3A). In average, 32% (± 13%) of neutrophils from COVID-19 patients expressed CD47 in comparison to 25% (± 15%) in healthy donors (*p* < 0.05; Fig. 3B). The expression of CD36 which is an “eat me” signal was not significantly different between groups (Fig. 3C–D).

### Low expression of CD49 on Neutrophils in COVID-19 patients indicates increased migration into lung tissue

**Figure 4:**
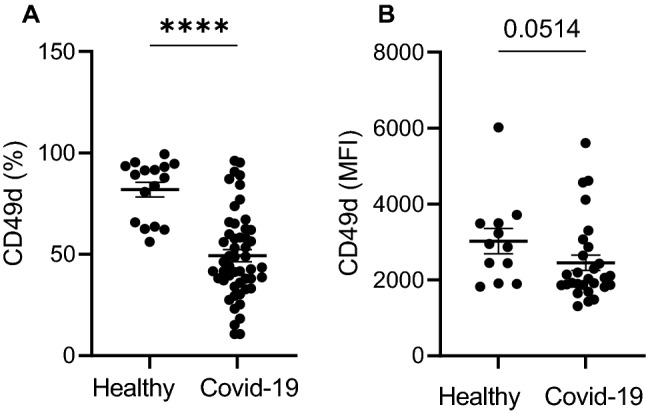
Neutrophils from COVID-19 patients present with significantly lower expression of CD49d compared with healthy individuals (**A**,**B**).

CD49^+^ neutrophils accumulate in the lung during viral infections^12^. Expression of CD49 is also increased on the surface of aged neutrophils. COVID-19 patients had approximately 50% CD49^+^ neutrophils in the blood as opposed to healthy controls, where over 80% of neutrophils in the blood expressed CD49 (49.3 ± 21.4 vs. 81.9 ± 14.6; *p* < 0.0001; Fig. 4A). MFI of CD49 on neutrophils was also lower compared with healthy controls. However, this finding did not reach statistical significance (*p* = 0.051, Fig. 4B). Low expression of CD49 on neutrophils in the blood indicates that neutrophils during SARS-CoV-2 infection migrate in high extent to peripheral tissues, including lungs, and that they are of young age.

### COVID-19 patients have high levels of NETs markers in their plasma

**Figure 5:**
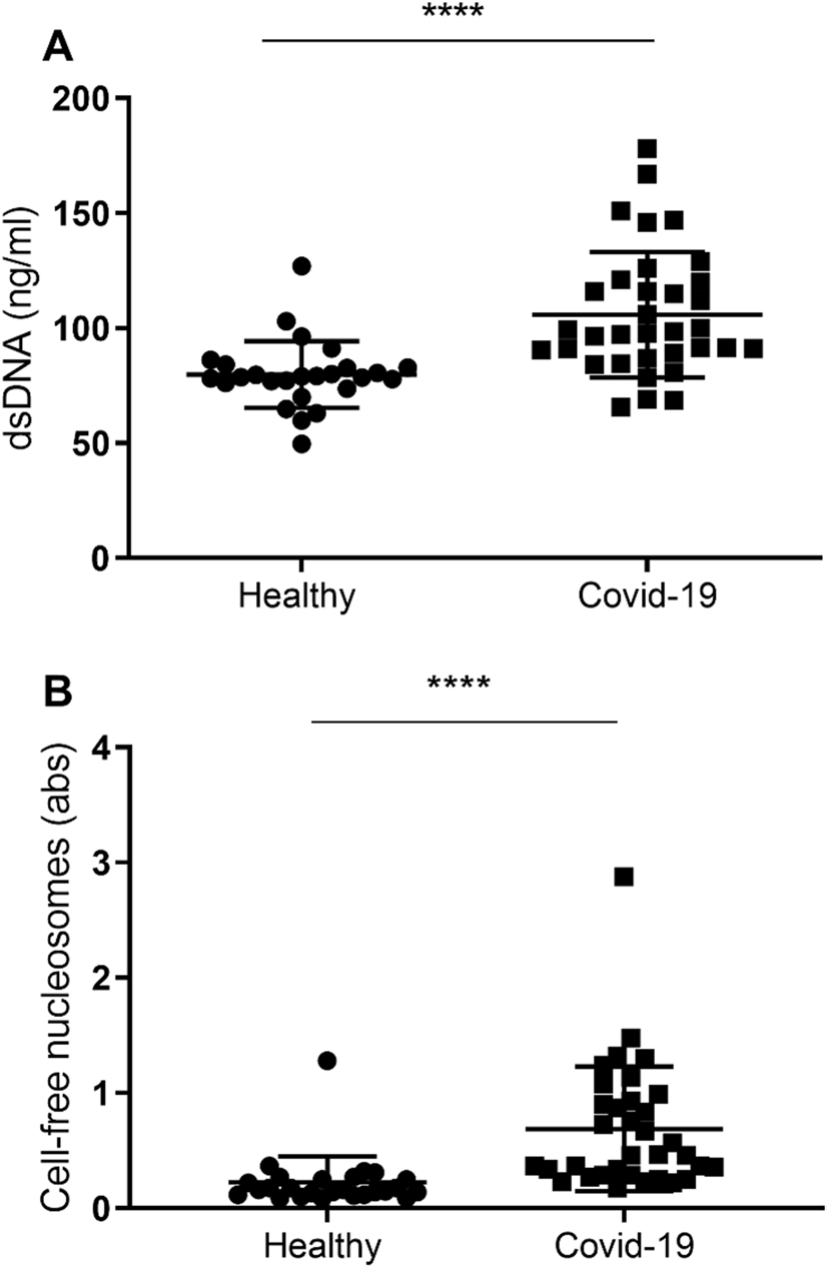
Plasma of COVID-19 patients shows increased levels of dsDNA (A) and cell free nucleosomes—markers of NETosis.

In line with previous research, plasma levels of circulating cell-free DNA (cf-DNA) and cell-free nucleosomes were significantly higher in patients with COVID-19 compared to healthy individuals (median (IQR) of 98.2 (88.7–120.3) vs. 78.9 (75.6–83.3); *p* < 0.0001 and 0.47 (0.29–0.95) vs. 0.16 (0.12–0.26); *p* < 0.0001; Fig. 5A–B). Expression of those markers indicates NETs formation in blood of affected patients.

### High-dimensional cluster analysis reveals neutrophil clusters characterized by long-lasting hyperreactivity

**Figure 6:**
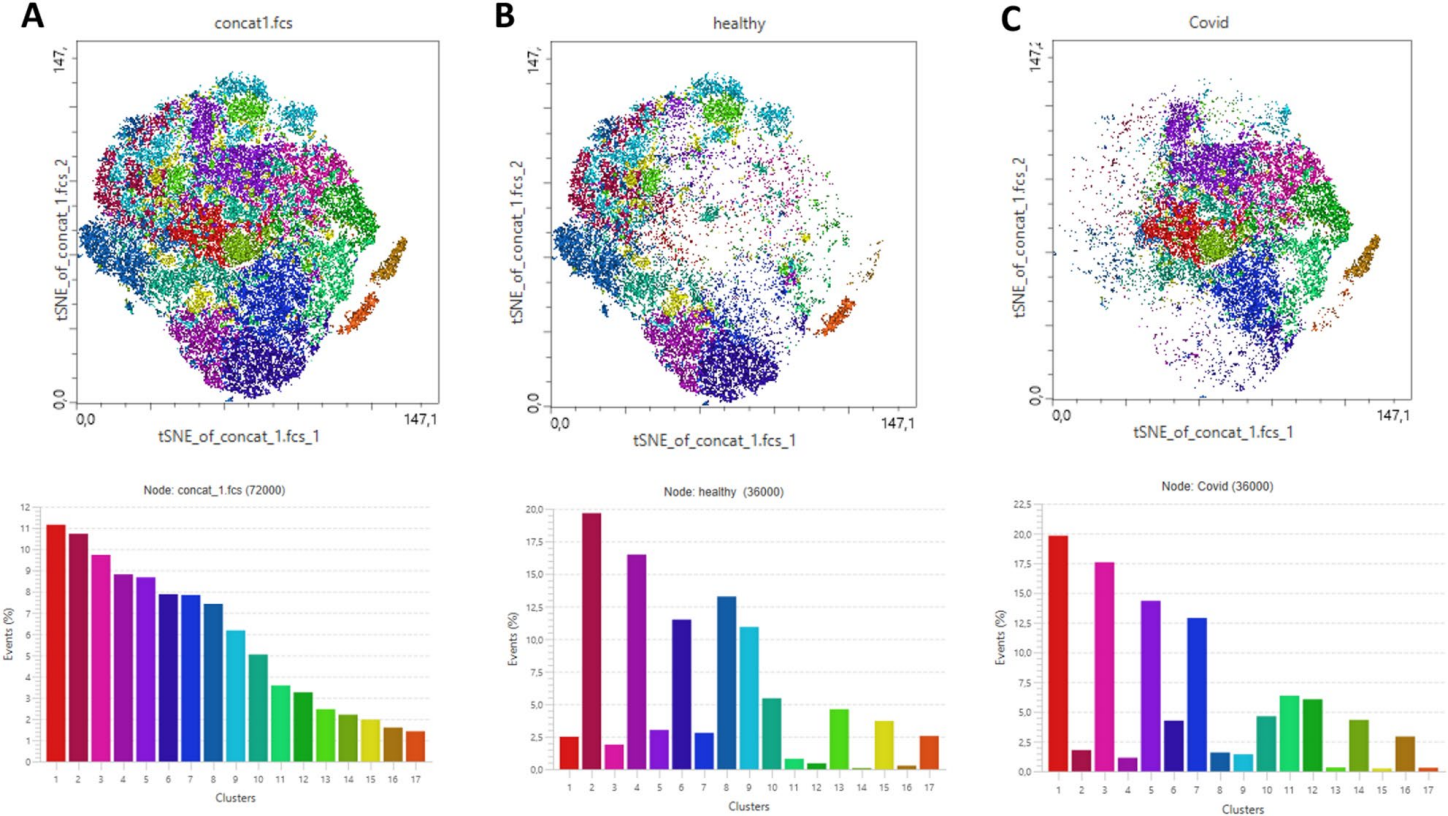
t‐SNE plots generated after data concatenation with hierarchical clustering of expression intensity (z score) for each of the indicated markers in each cluster derived using Phenograph. (**A**) Overview of all 17 clusters delineated within concatenated data for all analyzed samples. (**B**) Phenograph‐derived cluster pattern in healthy individuals. (**C**) Phenograph‐derived clusters pattern in COVID-19 patients. COVID-19 associated neutrophils were predominantly localized in clusters 1, 3, 5, 7, 10–12, 14 and 16. Neutrophils from healthy controls were grouped within clusters 2, 4, 6, 8, 9, 13, 15 and 17.

To extend findings, we processed data as described in the Methods section and mapped the neutrophil populations on t‐SNE composite plots, which revealed clear localizations of neutrophil populations in COVID-19 patients and healthy controls. Phenograph analysis revealed 17 unique clusters in the t‐SNE space. The detailed characterization of clusters is attached as Supplementary table 1. Figure 6 shows the distribution and localization of neutrophils in all study participants (Fig. 6A), COVID-19 patients only (Fig. 6B) and healthy controls only (Fig. 6C).

As can be seen in Fig. 6, the neutrophil populations in COVID-19 patients are characterized by completely opposite phenotypes than the populations seen in healthy controls. Neutrophils from healthy controls were grouped within clusters 2, 4, 6, 8, 9, 13, 15 and 17 (Fig. 6B). COVID-19 associated neutrophils were predominantly localized in clusters 1, 3, 5, 7, 10–12, 14 and 16 (Fig. 6C). Clusters from patients infected with SARS-CoV-2 included neutrophils characterized by high expression of activation markers (CD66b, CD11b, CD62L) and “don’t eat me” markers (CD47).

**Figure 7:**
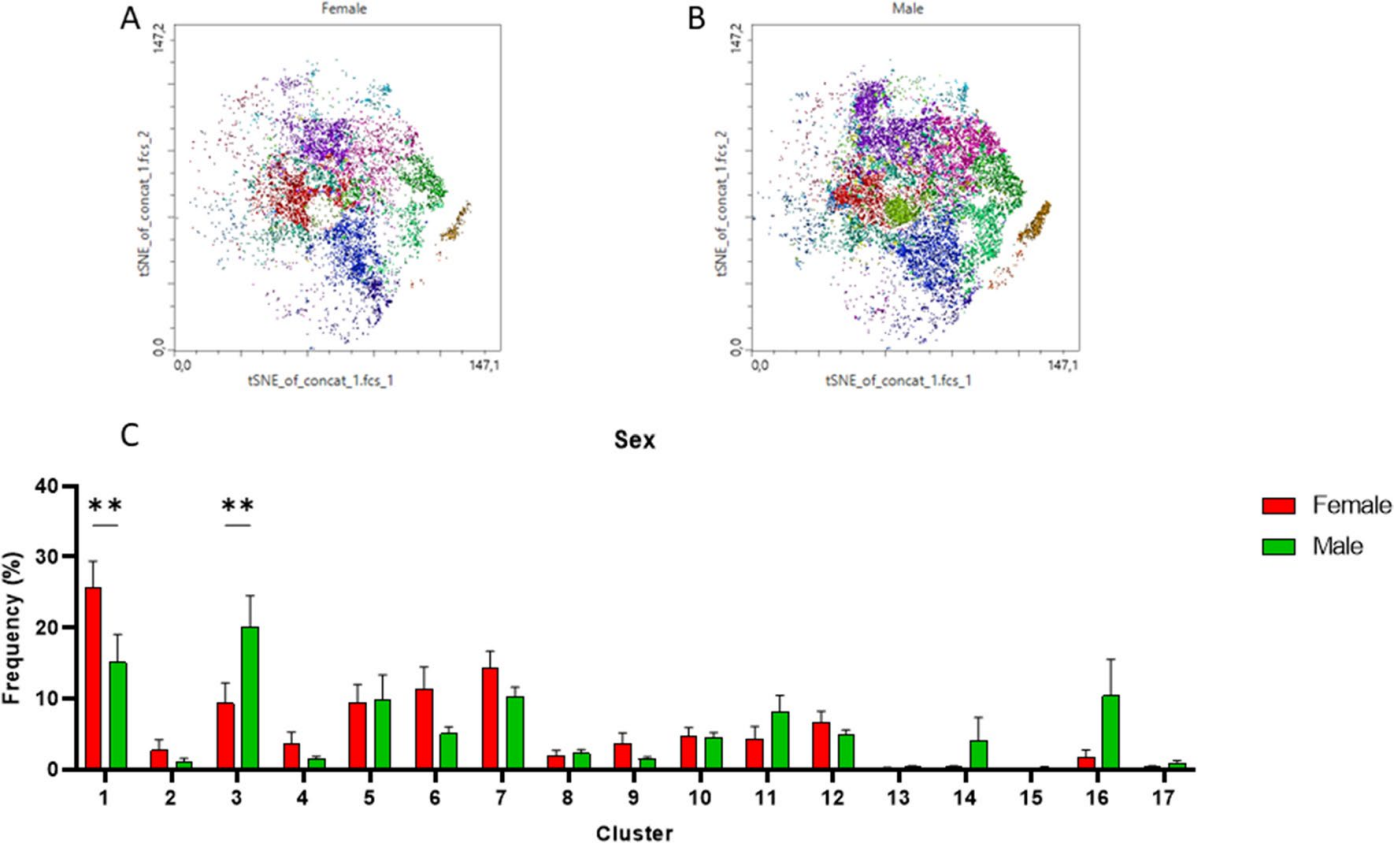
Comparison of Phenograph‐derived cluster pattern of neutrophils in (**A**) female and (**B**) male patients during COVID-19. Percentage distribution of clusters in female and male shown in a bar graph (**C**). Male patients had significantly more neutrophils plotted in cluster 3 (*p* < 0.01) that includes highly activated neutrophils, whereas women had significantly more neutrophils plotted within cluster 1 that includes mature non-activated neutrophils (CD16^high^CD62L^high^).

Neutrophil populations from COVID-19 patients were plotted on the same t-SNE patterns in regard to sex (Fig. 7A–C). The analysis revealed that male patients compared to female patients had significantly more neutrophils plotted in cluster 3 (*p* < 0.01) that includes neutrophils with high expression of “don’t eat me” and activation markers (CD47, CD11b). On the other side, women had significantly more neutrophils plotted within cluster 1 that includes mature non-activated neutrophils (CD16^High^CD62L^High^).

**Figure 8:**
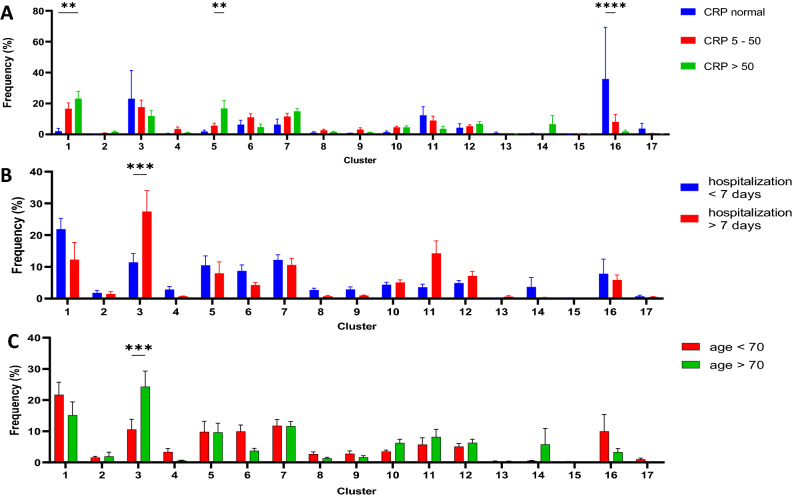
Comparison of Phenograph‐derived cluster pattern of neutrophils in (**A**) patients with normal level of CRP, CRP between 5 and 50 mg/L and CRP over 50 ; in (**B**) patients hospitalized shorter than 7 and longer than 7 days; in (**C**) patients younger than 70 year and older than 70 years. Older patients and those with longer hospitalization time had significantly higher percentage of neutrophils within cluster 3 that represents neutrophils with high expression of “don’t eat me” and activation markers such as CD11b and CD66b.

Furthermore, patients with markers of hyperinflammation in peripheral blood (high levels of C-reactive protein CRP and high Ferritin level) were characterized by overrepresentation of neutrophils within clusters 5 (representing pre-mature non-activated neutrophils) and 1 (representing mature non-activated neutrophils). On the other side, those patients with normal level of CRP had significantly more neutrophils that can be classified as aged and pre-apoptotic (Fig. 8A).

Another analysis, comparing patients with severe disease defined as hospitalization time over 7 days and milder disease with hospitalization time under 7 days, revealed that cluster 3 including hyperactivated neutrophils expressing “don’t eat me” markers are overrepresented in patients with severe disease (Fig. 8B) as well as in older patients (over 70 years old) (Fig. 8C).

## Discussion

The COVID-19 pandemic has a serious impact on global health and healthcare systems worldwide. Despite enormous efforts and successful development of vaccines, there are still hundreds of thousands of people getting infected by SARS-CoV-2 virus and developing sever acute respiratory failure across the globe. There is an urgent need to develop new therapies to treat the infection in those who did not have a chance to get vaccinated or despite vaccination become sick. However, drug discovery will not progress without profound knowledge about the pathophysiology of COVID-19 disease caused by SARS-CoV-2 virus. Accumulating evidence show that a major reason for development of severe COVID-19 is the hyperreactivity of the immune system leading to Acute Respiratory Distress Syndrome (ARDS) and neutrophils are being pointed out as one of the main drivers of this hyperinflammation^13,14^.

In this project, we used multicolor flow cytometry and ELISA to investigate in depth the phenotypes and biology of neutrophils under infection with SARS-CoV-2 virus. The data presented here provides an overview of activation patterns, survival and migration patterns of neutrophils from patients who were hospitalized due to COVID-19. Our data showed that neutrophils in COVID-19 patients are hyperactivated, have significantly prolonged survival, produce in high extent NETs and migrate into peripheral tissues—likely into lungs. Finally, our investigation identified a novel neutrophil phenotype that correlated with severity of the disease, defined as length of hospitalization over 7 days, and poor prognosis predictors—namely, age over 70 and male sex.

Neutrophils are the most abundant immune cells in humans. They have for long been considered as fairly simple cells belonging to the innate immune system, which predominant function is a direct, unspecific response to bacterial and fungal pathogens. The antiviral immune responses are on the other hand claimed to be driven by predominantly the adaptive immune system. Nevertheless, multiple studies show that neutrophils play an important role in viral infections from e.g. herpes simplex virus (HSV), influenza virus and respiratory syncytial virus^15–17^. To fight against viral pathogens, neutrophils possess a variety of biological mechanisms such as direct phagocytosis of viruses and production of cytokines, chemokines and NETs.

Neutrophils have been shown to increase in numbers during COVID-19 infection together with a decreased count of T lymphocytes^1^. Furthermore, the elevated neutrophil-to-lymphocyte ratio (NLR) was associated with poor prognosis^8^. Markers of neutrophil extracellular traps (NETs) were also shown to be elevated in COVID-19 patients and correlated with poor prognosis^18^. Our study contributes to the knowledge of neutrophil biology during infection with SARS-CoV-2, by showing a significantly increased survival of circulating neutrophils in infected patients. High expression of CD47 (“don’t eat me” marker) has not been shown before in patients with COVID-19. The increased survival of neutrophils can prolong the duration of negative effects of those cells on the immune system and drive neutrophile-associated tissue damage, especially in the lungs. Our findings support the theory that neutrophils with deviating phenotypes may be responsible for driving the hyperinflammation following infection with SARS-CoV-2. Further, it is confirmed by high levels of neutrophil-associated pro-inflammatory cytokines such as TNFα, IL-6 and IL-8.

In light of recent findings, neutrophils seem to be a natural target for potential novel therapeutic developments. One of the well-known and already used pharmacological agents for treatment of severe COVID-19 are corticosteroids that were shown to reduce the recruitment of neutrophils and decrease the neutrophil associated hyperinflammation^19^. However, since corticosteroids influence several cells and organs and have a variety of systemic side effects, there is a need for further research and development of therapeutics targeting predominantly neutrophils. There are currently some ongoing clinical trials focusing on blocking the neutrophil functions to prevent tissue damage in the lungs^20^. One of the potential drugs being tested, anakinra (IL1R inhibitor), showed promising preliminary results^21,22^. Other studied targets include the complement C5a-C5aR1 interaction^23^ or CXCL8/CXCR2 axis that is responsible for neutrophil migration^11^.

However, this study is not without limitations. It is important to emphasize that it is a single-center study, conducted on a relatively small number of patients. Further, our cohort of COVID-19 patients was to some extent heterogeneous in regard to age and comorbidities. The population of COVID-19 patients studied in this project had a significantly higher median age compared to the healthy controls. The younger age of the controls is the result of the COVID-19 recommendations in Region Stockholm, which advocated for avoiding unnecessary contact of the elderly with the health care system during the pandemics. Therefore, only younger volunteers were recruited. It is known that the activation of the neutrophils in response to pathogens declines in elderly people^24^. Thus, an age difference could be a potential confounding factor. Nevertheless, our findings show a significantly higher activation and responsiveness of neutrophils in the COVID-19 population which was older indicating that the activation in younger patients can be expected to be even higher. Moreover, we included only patients in need of hospitalization due to their COVID-19, therefore findings of this project depict the neutrophils phenotypes in moderate and severe COVID-19. By studying this cohort, we were not able to characterize the neutrophils in patients with mild COVID-19.

## Conclusion

With this study, we are highlighting the important role of neutrophils in the pathophysiology of COVID-19. Neutrophils from patients infected with SARS-CoV-2 have increased survival and predominantly express a phenotype associated with hyperinflammation, which is believed to be the driver of severe COVID-19. Furthermore, we identified a novel subset of neutrophils that are predominantly found in those patients who need a longer hospitalization time. Those neutrophils were characterized by high expression of “don’t eat me” and activation markers such as CD11b and CD66b. Here we challenge, once again, the hallmark of immunology showing that neutrophils are neither homogenous nor a short-lived population of cells. Our findings are a step towards better understanding of neutrophil functions not only during COVID-19 but also in many other viral, bacterial and inflammatory conditions.

## Supplementary Information

Supplementary Information 1.

Supplementary Information 2.

## Data Availability

All data generated or analyzed during this study are included in this published article and its supplementary information files.

## Abbreviations

NETs: Neutrophil extracellular traps
NLR: Neutrophil-to-lymphocyte ratio
ARDS: Acute respiratory distress syndrome
