# Supplementary material for: A prolonged innate systemic immune response in COVID-19
Source: Sci Rep. 2022 Jun 15;12:9915. doi: 10.1038/s41598-022-13986-5 (PMC9200737; doi:10.1038/s41598-022-13986-5)
Supplement: Supplementary file 1 — Supplementary Information 1. [file 41598_2022_13986_MOESM1_ESM.docx]

Manuscript title: A prolonged innate systemic immune response in COVID-19

Supplementary Table 1. Characterization of expression markers in Phenograph‐derived clusters.

1. CD36-CD11+/-CD43+CD16+CD62L+CD49+/-CD47+/-CD24+/- CD66b+/-
2. CD36-CD11-CD43+CD16+CD62L+CD49+/-CD47-CD24- CD66b-
3. CD36-CD11+CD43+CD16+CD62L-CD49-CD47+/-CD24+/- CD66b-
4. CD36+CD11-CD43+CD16+CD62L+CD49+CD47+/-CD24+/- CD66b-
5. CD36-CD11-CD43+CD16+CD62L+CD49--CD47-CD24- CD66b-
6. CD36+CD11+CD43+CD16+CD62L+CD49+CD47+CD24+/- CD66b-
7. CD36+CD11+/-CD43+CD16+CD62L+CD49-CD47+CD24+/- CD66b-
8. CD36-CD11-CD43+CD16+/-CD62L+CD49+CD47-CD24+/- CD66b-
9. CD36-CD11-CD43+CD16+CD62L-CD49+/-CD47-CD24+/- CD66b-
10. CD36-CD11+/-CD43+CD16+CD62L+CD49+CD47+CD24+/- CD66b-
11. CD36+CD11+CD43+CD16+CD62L-CD49+/-CD47+CD24+/- CD66b+
12. CD36-CD11+CD43+CD16+CD62L-CD49++CD47+CD24- CD66b+
13. CD36-CD11+/-CD43+CD16++CD62L+/-CD49-CD47--CD24-CD66b-
14. CD36+CD11+/-CD43+CD16-CD62L+/-CD49+/-CD47+/-CD24+/- CD66b+
15. CD36-CD11+/-CD43+CD16+/-CD62L+/-CD49-CD47-CD24+ CD66b-
16. CD36-CD11-CD43+CD16--CD62L--CD49+/-CD47+/-CD24+/- CD66b+
17. CD36+CD11+/-CD43+CD16--CD62L+/-CD49+/-CD47+CD24+ CD66b+


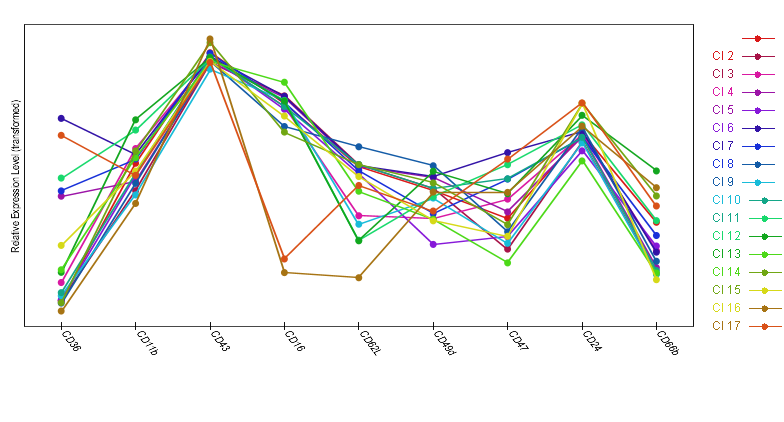


Cl 1
