# Supplementary material for: A prolonged innate systemic immune response in COVID-19
Source: Sci Rep. 2022 Jun 15;12:9915. doi: 10.1038/s41598-022-13986-5 (PMC9200737; doi:10.1038/s41598-022-13986-5)

Supplementary Figure 1. The gating strategy for the analysis of neutrophils in PBMC is delineated by an exemplary dot plot. (A) First, neutrophils were selected based on SSC-A and FSC-A parameters and used for further analysis. Secondly, doublets were excluded (B) and neutrophils were detected based on the expression of CD15 (C).


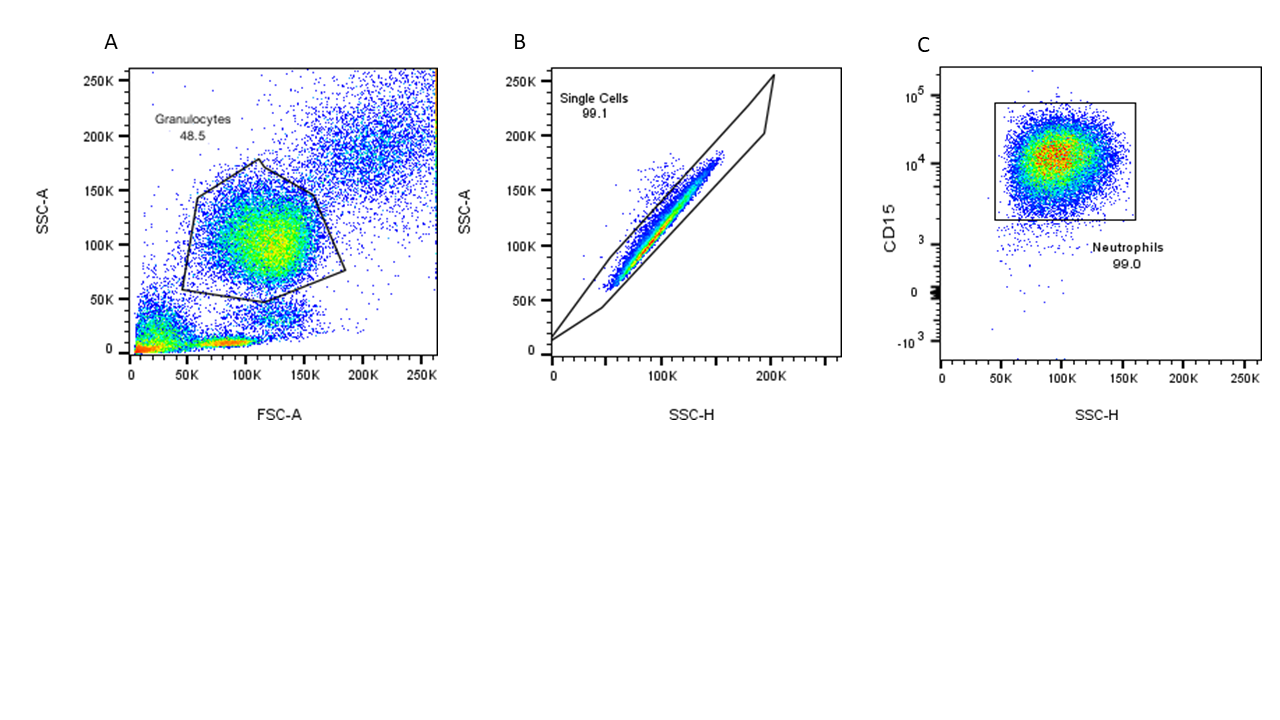

Supplement: Supplementary file 2 — Supplementary Information 2. [file 41598_2022_13986_MOESM2_ESM.docx]
